# Narrow-based gait in people with Parkinson's disease: Its mechanisms explored

**DOI:** 10.1177/1877718X241313333

**Published:** 2025-02-02

**Authors:** Jamie AF Jansen, Tom JW Buurke, Lotte van de Venis, Vivian Weerdesteyn, Noël Keijsers, Jorik Nonnekes

**Affiliations:** 1 Radboud University Medical Center; Donders Institute for Brain, Cognition and Behavior; Department of Rehabilitation, Center of Expertise for Parkinson & Movement Disorders, Nijmegen, The Netherlands; 2 Department of Human Movement Sciences, University of Groningen, University Medical Center Groningen, Groningen, The Netherlands; 3 Department of Movement Sciences, KU Leuven, Leuven, Belgium; 4 Department of Research, Sint Maartenskliniek, Nijmegen, The Netherlands; 5 Department of Sensorimotor Neuroscience, Radboud University, Donders Institute for Brain, Cognition and Behaviour, Nijmegen, The Netherlands; 6 Department of Rehabilitation, Sint Maartenskliniek, Ubbergen, The Netherlands

**Keywords:** Parkinson’s disease, gait, balance, center of mass, margin of stability

## Abstract

**Background:**

People with Parkinson's disease (PD) typically exhibit a narrow-based gait. We previously found that walking with reduced trunk rotation and obliquity led to narrow-based gait in healthy adults; a decrease in trunk motion coincided with a decrease in mediolateral extrapolated center of mass (XCoM) excursion, requiring a smaller step width to maintain a constant mediolateral margin of stability (MoS).

**Objective:**

To assess whether reduced trunk motion in PD is related to narrow-based gait, without affecting mediolateral MoS. To explore the underlying mechanisms of narrow-based gait, we examined the effects of increasing arm swing (aiming to increase trunk motion), and widening steps on gait in PD.

**Methods:**

Fifteen people with PD and narrow-based gait and 17 age-matched controls walked on a treadmill for three minutes at a fixed gait speed during three conditions: baseline, increased arm swing and widened step width. Step width, trunk rotation and obliquity were calculated using marker data, and XCoM excursion and MoS using ground reaction forces.

**Results:**

Trunk rotation, XCoM excursion, and step width were significantly smaller in PD compared to controls, while the MoS did not differ. Increased arm swing did not substantially increase trunk motions in PD, though people with PD were able to widen their step width.

**Conclusions:**

We provide further evidence for a relation between trunk motion and step width. In PD, reduced trunk motion may contribute to narrow-based gait, without affecting mediolateral MoS; future work is needed to confirm a causal relationship between reduced trunk motion and narrow-based gait in PD.

## Plain language summary

People with Parkinson's disease (PD) typically walk with a smaller base of support compared to healthy adults. The underlying mechanisms are unclear. In an earlier study that we conducted among healthy adults, we found that walking with reduced trunk motion led to a narrow-based gait pattern, without affecting sideways stability. In this study, we tested whether reduced trunk motion is related to narrow-based gait in people with PD, while having no impact on sideways stability. To further explore the mechanisms underlying narrow-based gait, we examined the effects of walking with increased arm swing (to increase trunk motion), and widening steps on gait in PD. This study included 15 people with PD, and 17 age-matched controls who walked on a treadmill for three minutes at a fixed gait speed during three experimental conditions. The conditions included: walking with no additional instructions, walking with increased arm swing, and walking with increased step width. The study variables collected were step width, trunk rotation and obliquity (as measures for trunk motion), center of mass excursions and a measure for (dynamic) stability. We found that trunk rotation, center of mass excursions and step width were smaller in people with PD compared to controls while stability did not differ. Increased arm swing did not lead to increased trunk motion in PD. People with PD were able to widen their step width. We have provided further evidence for a relation between trunk motion and step width. In people with PD, reduced trunk motion may contribute to narrow-based gait, without affecting stability. Future studies should confirm the relationship between reduced trunk motion and narrow-based gait in PD.

## Introduction

Gait impairments are a disabling symptom in people with Parkinson's disease (PD) and impact daily activities and quality of life.^1–3^ Gait impairments in PD involve a slow walking speed, a reduced step length and step height, a stooped posture, and asymmetrically reduced arm swing.^4^ Moreover, patients typically exhibit a narrow-based gait,^4–6^ although a widened base of support can also be seen in patients with co-morbidities.^7^ At first sight, it may be counterintuitive that people with PD typically narrow their step width instead of adopting a widened base of support, a pattern commonly seen in other neurological disorders, such as cerebellar ataxia and normal pressure hydrocephalus.^4^

The mechanisms underlying narrow-based gait in PD are yet to be unraveled.^4,8–10^ It has been suggested that people with PD unconsciously apply a narrowed base of support to compensate for a reduction in amplitudes of anticipatory postural adjustments (APAs); lateral weight shifts towards the stance leg to unload the subsequential swing leg during walking.^9,10^ However, the observation of broad-based gait in PD patients with co-morbidities argues against this notion.^4,11^ Recently, our team hypothesized that narrow-based gait may arise from reduced trunk motion that is typically observed in PD (potentially due to trunk rigidity). Trunk motion can be defined as a combination of trunk rotation (i.e., movement in the sagittal axis of the trunk) and obliquity (i.e., movement in the transverse axis of the trunk).^12^ In support of this hypothesis, it was found that walking with reduced trunk motion resulted in narrow-based gait in healthy adults.^8^ Biomechanically, during walking, humans mainly control their center of mass (CoM) via foot placement, which in turn determines step width.^13^ The movement of the CoM and mediolateral stability during gait can be expressed by the extrapolated center of mass (XCoM, i.e., taking into account the CoM position and its velocity) and mediolateral margin of stability (MoS, i.e., the distance between the mediolateral XCoM and mediolateral base of support).^14–16^ It has been postulated that people without balance impairments aim for a constant offset of the MoS when environmental demands are stable.^14,15^ Reduced trunk motions would coincide with decreased mediolateral XCoM excursions, and this would require a smaller step width to maintain a similar MoS.^8,14,15^ Thus, if people with PD exhibit smaller mediolateral XCoM excursions due to reduced trunk motion, a smaller step width will result in a similar mediolateral MoS compared to healthy controls and, therefore, a stable yet narrow-based gait pattern.

Here, we assessed whether reduced trunk motion in PD is related to narrow-based gait without affecting the mediolateral MoS. We hypothesized that trunk motion and mediolateral XCoM excursions are reduced in people with PD who exhibit a narrow-based gait, but that the mediolateral MoS is similar to age-matched healthy controls. To gain further insight into the underlying mechanisms of narrow-based gait in PD, we aimed to experimentally increase trunk motion, by having participants walk with increased arm swing. We hypothesized that increased arm swing would considerably increase trunk motion and consequently enlarge mediolateral XCoM excursion and step width while having no effect on mediolateral MoS. Finally, we verified whether patients are able to broaden their step width, which we hypothesized to be the case.

## Methods

### Participants

In this experimental study, we included 15 people with idiopathic PD and 17 age-matched healthy controls. Baseline patient characteristics can be found in Table 1. Inclusion criteria for people with idiopathic PD involved the ability to walk on a treadmill for ten minutes and follow instructions, and having a narrow-based gait pattern (also on a treadmill), as classified by the eye of a movement disorders expert. During screening, we excluded three PD subjects with a normal-based gait. Moreover, patients with comorbidities that impacted gait or balance capacity were excluded. All participants provided written informed consent before participating in this study, in agreement with the Declaration of Helsinki^17^ and the local ethical regulations (Medical Ethical Committee Arnhem-Nijmegen dossier ‘2021-7497’).

**Table 1.** Baseline patient characteristics.

	Parkinson's Disease n = 15	Healthy Controls n = 17	*p*
Age (y)	69 ± 7.2	65 ± 10.69	0.261
Men (n, %)	9 (60%)	11 (65%)	0.761
Disease duration (y)	9.1 ± 4.62	N/A	N/A
MDS-UPDRS Part III (median, range)	42 (8–80)	N/A	N/A
Fallen in the past year (n, %)	8 (53%)	1 (6%)	0.005
Activity Balance Confidence scale percentage (mean, range)	68 (30–93)	93 (71–100)	<0.001
MiniBest total score (median, range)	20 (8–26)	27 (21–28)	<0.001

### Experimental protocol

Assessments took place at the Radboud University Medical Center (Radboudumc, Nijmegen, the Netherlands). People with PD were measured in the dopaminergic ON-state (measurements started 30 min after intake of their regular dosage of dopaminergic medication). For all participants, we collected information on demographics and the presence of falls in the past year. Balance confidence during ambulatory activities was determined by the activity-specific balance confidence scale (ABC-scale),^18^ and balance capacity was tested using the mini-BESTest.^19^ Additionally, in people with PD, we assessed disease severity by the MDS-UPDRS part III.^20^

Subsequently, participants were asked to walk on an instrumented dual-belt treadmill (Motek, Amsterdam, NL) while wearing a safety harness attached to the ceiling that did not influence movement or support the participant's body weight. Participants walked on a dual-belt treadmill at a preset pace of 0.6 ms^−1^ during three conditions, each lasting three minutes. In the first condition, participants walked without additional instructions (*baseline condition*). In the second condition, participants were instructed to exaggerate their arm swing in antiphase to their natural step rhythm, while no comments on trunk movements or step width were made (*arm swing condition*). In the third condition, with the goal to assess the ability to widen their step width, a 15 cm beam was projected on the middle of the treadmill. In this condition, participants were instructed to increase their step width by positioning their feet outside the projected beam (*beam condition*). This means that in order to step outside of the projected beam, people had to increase their step width (difference in mediolateral CoP position between toe-offs) significantly more than 15 cm. We encouraged the participants to focus on stepping outside the beam when their step width narrowed during the trial. During all trials, participants were instructed to look forward as much as possible while walking.

### Data acquisition

3D kinematics were captured using a 10-camera motion capture system (Vicon Motion System Ltd, Oxford, UK) at 100 Hz. Markers were placed on the participant according to the Plug-in Gait full-body model.^12^ 6D ground reaction forces and moments (N, Nm) and 2D center of pressure positions (m) were measured using the treadmill's embedded force plates at 2000 Hz. Raw data were preprocessed in Vicon Nexus (Vicon Motion Systems Ltd, Oxford, UK).

### Data analysis

Processed data were analyzed in Matlab 2022a (MathWorks Inc., Natick, MA, USA). Gait events, i.e., time points of initial contacts and toe-offs, were determined by localizing local peaks and valleys in the anteroposterior direction of the foot marker data.^21^ During treadmill walking, peaks represent the timepoints when the foot contacts the treadmill, whereas the valleys indicate the instances when the foot lifts off. Trunk motion (i.e., rotation and obliquity) was obtained from the Vicon Plug-in gait model.^12^ Maximum range of trunk motion (degrees) was calculated as the difference between the lowest and highest values within a stride. Trunk rotation (degrees) was calculated as trunk axial rotation according to the recommendations of the international society for biomechanics.^22^ Trunk axial rotation to the left is defined as positive and to the right as negative. Trunk obliquity was calculated as trunk lateral rotation. Trunk lateral flexion rotation to the right is positive, to the left negative. A low-pass 15 Hz 2^nd^ order Butterworth filter was used to filter ground reaction forces and center of pressure (CoP) positions.^23,24^ Step width was defined as the difference in the mediolateral CoP position between ipsi- and contralateral toe-off for each step.^8,15^ Cadence was defined as the number of steps per minute and is inversely proportional to step length, when walking at a fixed gait speed.

Mediolateral CoM position (m) was determined by combining the twice-integrated and high-pass filtered (0.2 Hz) mediolateral GRF divided by the subject's weight with the low-pass filtered (0.2 Hz) mediolateral center of pressure.^23–26^ The mediolateral XCoM was determined as in Eq.1.^15^ The difference in mediolateral XCoM position between ipsilateral and contralateral toe-off for each stride defined the mediolateral XCoM excursion (m). MoS (m) was determined by the minimal distance between the mediolateral CoP position and the mediolateral XCoM position during the single support phase of each step.^15,16^

[Eq.1] $$\begin{array}{rl} & XCoM=CoM+\frac{vCoM}{\sqrt{\frac{g}{l}}},\;\mathrm{in}\;\mathrm{which}\;\\ & \;\;g=9.81\;m\;s^{-2},\;\mathrm{and}\\ & \;\;l=\mathrm{effective}\;\mathrm{pendulum}\;\mathrm{length}\;(1.2*\mathrm{trochanteric}\;\mathrm{height}). \end{array}$$

In the arm swing condition, we first verified whether people swung their arms in phase with the contralateral leg movement. We obtained the arm swing patterns (i.e., shoulder flexion: the angle between the sagittal-humerus and the sagittal-thorax around a fixed transverse axis) from the Vicon Plug-in gait model.^12^ To verify whether people swung their arms in phase with the contralateral leg, we determined whether the maximal degree of arm swing occurred within a 40% window, (i.e., 20% before or after) of the moment of contralateral initial contact. We isolated and analyzed data of at least ten consecutive steps in which arm swing fulfilled these criteria. Data from two patients had to be excluded because this never happened during the entire trial.

### Statistical analysis

Statistical analysis was performed using IBM SPSS 27 (SPSS, Inc, Chicago, IL). Group characteristics and the mean baseline gait variables of interest, i.e., step width, XCoM, MoS, trunk rotation, trunk obliquity and cadence, were tested for normality using the Shapiro-Wilk test and compared between the PD group and healthy controls using an independent samples T-test or the non-parametric independent samples Mann-Whitney U test. We subsequently performed a paired samples T-test or a related-samples Wilcoxon signed rank test. In both controls and PD, we compared the mean arm swing, trunk rotation, trunk obliquity, XCoM, step width and MoS of the baseline condition to the arm swing condition in order to determine the effects of increased arm swing. In addition to investigate the relationship between the change of parameters between the baseline and the arm swing condition, we determined the Pearson correlation coefficients of the change of mean trunk rotation, obliquity, XCoM, step width and MoS. In addition, to show that people with PD could widen their step width, we compared the mean step width in the baseline condition to the mean step width in the beam condition.

## Results

**Figure 1.**
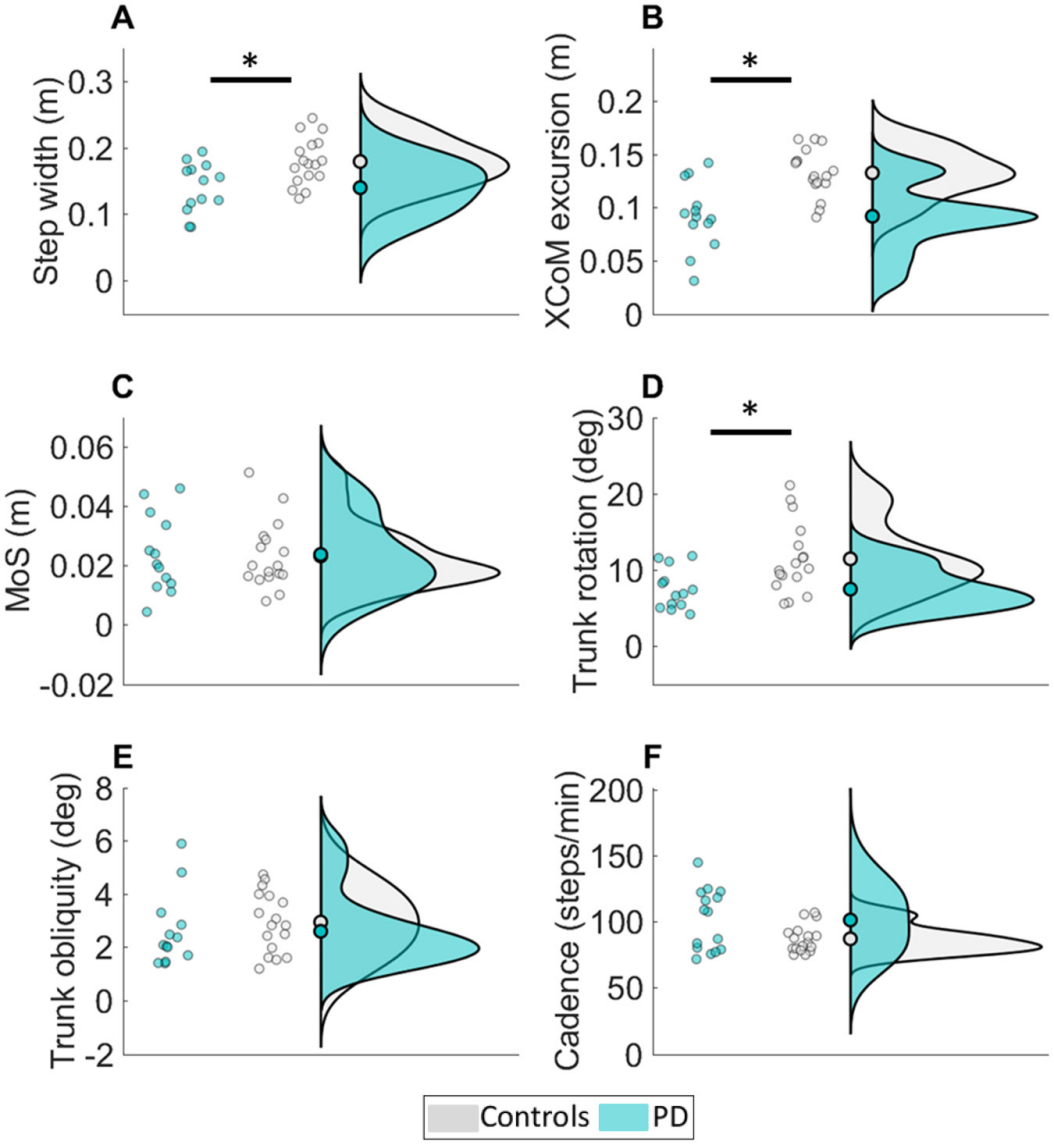
Comparison of the baseline condition (walking at a fixed speed of 0.6 m s^−1^) between people with PD (turquoise) and healthy controls (white). People with PD had a more narrow-based gait and reduced extrapolated center of mass (XCoM) excursions, while maintaining a similar margin of stability (MoS). Trunk rotation, but not trunk oblique was significantly smaller in people with PD. There was no difference in cadence between groups.

In the baseline condition, all participants were able to walk at 0.6 m s^−1^. As per our inclusion criterium, step width (Table 2; Figure 1A) was smaller in people with PD (14.1 ± 3.9 cm)) compared to controls (18.0 ± 3.5 cm); t_(30) = _2.941, p = 0.006). Mediolateral XCoM excursion (Figure 1B) was smaller in people with PD (9.0 ± 3.1 cm) compared to controls (13.3 ± 2.2 cm); t_(30) _= 4.517, p < .001), while the mediolateral MoS (Figure 1C) did not differ between both groups (PD: 2.5 ± 1.3 cm, controls: 2.3 ± 1.1 cm; t_(30) _= -0.377, p = 0.709). Trunk rotation (Figure 1D) was smaller in people with PD (7.7 ± 2.6 degrees) compared to controls (11.5 ± 4.6 degrees; t_(30) _= 2.796, p = 0.009), whereas trunk obliquity (Figure 1E) did not differ between both groups (PD: Mdn: 2.0 IQR: 1.4 degrees, controls: Mdn: 2.8 IQR: 2.2 deg; U = 92, p = 0.180). There was no significant difference in cadence (Figure 1F) between PD and controls (p = 0.142).

**Table 2.** Comparison of the baseline condition between people with PD and healthy controls. Values are represented as mean ± SD, unless otherwise specified.

	Parkinson's Disease n = 15	Healthy Controls n = 17	T-stats
Step width (cm)	14.1 ± 3.9	18.0 ± 3.5	t(30) = 2.941, p = 0.006
ML XCoM excursion (cm)	9.0 ± 3.1	13.3 ± 2.2	t(30) = 4.517, p < 0.001
ML MoS (cm)	2.5 ± 1.3	2.3 ± 1.1	t(30) = 0.377, p = 0.709
Trunk rotation (degrees)	7.7 ± 2.6	11.5 ± 4.6	t(30) = 2.796, p = 0.009
Trunk obliquity (Mdn, degrees)	2.0 IQR: 1.4	2.8 IQR: 2.2	U = 92, p = 0.180
Cadence (Mdn, steps/min)	108 IQR: 43	85 IQR: 13	U = 167, p = 0.142

Controls successfully increased arm swing in the arm swing condition (100.0 ± 14.0 degrees; Table 3, Supplemental Figure 1) compared to the baseline condition (17.2 ± 9.4 degrees; t_(16) _= 4.187, p=<0.001). Both trunk rotations (baseline: 11.5 ± 4.6 degrees, arm swing: 18.7 ± 10.0 degrees; t_(16) _= 3.937, p=<0.001) and trunk obliquity (baseline: Mdn: 2.8 IQR: 2.2 degrees, arm swing: Mdn: 3.1 IQR: 2.9 degrees; Z = 130, p = 0.011) increased in the arm swing condition. In addition, mediolateral XCoM excursions increased in the arm swing condition (15.4 ± 2.5 cm) compared to the baseline condition (13.3 ± 2.2 cm; t_(16) _= 4.859, p=<0.001). Step width also increased in the arm swing condition (baseline: 18.0 ± 3.5 cm, arm swing: 20.2 ± 3.5 cm; t_(16) _= 4.187, p=<0.001), while the mediolateral MoS remained similar to baseline (baseline: 2.3 ± 1.1 cm, arm swing: 2.4 ± 1.1 cm; t_(16) _= 0.364, p = 0.720).

In controls, the change in trunk rotation (r = 0.461, p = 0.063) and trunk obliquity (r = 0.231, p = 0.372) between the arm swing condition and the baseline condition were not correlated with the XCoM. Change in XCoM was correlated with the change in step width (r = 0.819, p < 0.001). Change in step width was correlated with the change in MoS (r = 0.552, p = 0.022).

**Figure 2.**
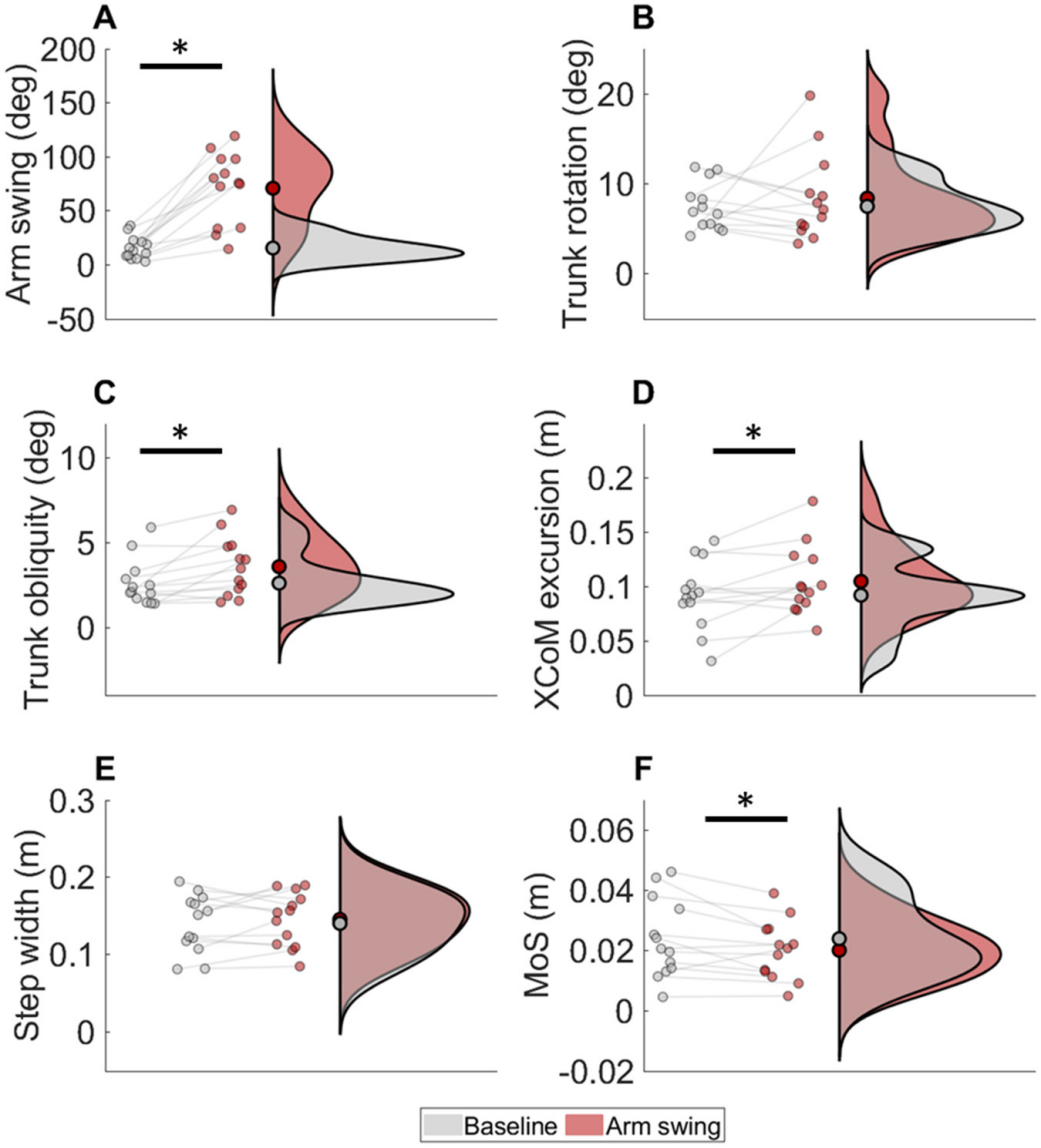
Comparison between baseline (grey) and the arm swing (red) condition in people with PD. People with PD were able to increase their degree of arm swing. Trunk rotation did not differ between conditions, but obliquity increased in the arm swing condition. Extrapolated center of mass (xCoM) excursions increased, but not step width. The margin of stability (MoS) remained similar.

People with PD successfully increased their arm swing (Table 3, Figure 2A) in the arm swing condition (71.0 ± 33.3 degrees) compared to the baseline condition (15.7 ± 10.5 degrees; t_(12) _= 6.765, p=<0.001). Trunk rotation (Figure 2B) did not differ between conditions (baseline: 7.5 ± 2.6 degrees, arm swing: 8.4 ± 4.8 degrees; t_(12) _= 0.582, p = 0.571), but trunk obliquity (Figure 2C) increased (baseline: Mdn: 2.1 IQR: 1.5 degrees, arm swing: Mdn: 3.5 IQR: 2.7 degrees; Z = 84, p = 0.007). Mediolateral XCoM excursions (Figure 2D) showed a small but significant increase in the arm swing condition (10.5 ± 3.2 cm) compared to the baseline condition (9.2 ± 3.2 cm; t_(12) _= 2.322, p = 0.039). Step width (Figure 2E) did not differ between conditions (baseline: 14.0 ± 3.8 cm, arm swing: 15.0 ± 3.5 cm; t_(12) _= 0.830, p = 0.423). Mediolateral MoS (Figure 2F) did differ in the arm swing condition (2.0 ± 1.0 cm) compared to the baseline condition (2.4 ± 1.3 cm; t_(12) _= 2.169, p = 0.040).

**Table 3.** Comparison of the baseline condition to the arm swing condition in healthy controls and people with PD. Values are represented as mean ± SD, unless otherwise specified.

	Baseline	Arm swing	T-stats
Healthy controls			
Arm swing (degrees)	17.2 ± 9.4	100.0 ± 14.0	t(16) = 4.187, p=<0.001
Trunk rotation (degrees)	11.5 ± 4.6	18.7 ± 10.0	t(16) = 3.937, p=<0.001
Trunk obliquity (Mdn, degrees)	2.8 IQR: 2.2	3.1 IQR: 2.9	Z = 130, p = 0.011
ML XCoM excursion (cm)	13.3 ± 2.2	15.4 ± 2.5	t(16) = 4.859, p=<0.001
Step width (cm)	18.0 ± 3.5	20.2 ± 3.5	t(16) = 4.187, p=<0.001
ML MoS (cm)	2.3 ± 1.1	2.4 ± 1.1	t(16) = 0.364, p = 0.720
People with PD			
Arm swing (degrees)	15.7 ± 10.5	71.0 ± 33.3	t(12) = 6.765, p=<0.001
Trunk rotation (degrees)	7.5 ± 2.6	8.4 ± 4.8	t(12) = 0.582, p = 0.571
Trunk obliquity (Mdn, degrees)	2.1 IQR: 1.5	3.5 IQR: 2.7	Z = 84, p = 0.007
ML XCoM excursion (cm)	9.2 ± 3.2	10.5 ± 3.2	t(12) = 2.322, p = 0.039
Step width (cm)	14.0 ± 3.8	15.0 ± 3.5	t(12) = 0.830, p = 0.423
ML MoS (cm)	2.4 ± 1.3	2.0 ± 1.0	t(12) = 2.169, p = 0.04

In PD, the change in trunk rotation (r = -0.068, p = 0.825) and trunk obliquity (r = 0.148, p = 0.630) between the arm swing condition and the baseline condition were not correlated with the XCoM. Change in XCoM (was correlated with the change in step width r = 0.873, p < 0.001). Change in step width was not correlated with the change in MoS (r = 0.528, p = 0.064).

People with PD were able to successfully increase their step width in the beam condition (26.8 ± 3.0 cm) compared to the baseline condition (14.1 ± 3.9 cm; t_(14) _= 9.470, p=<0.001).

## Discussion

This experimental study aimed to shed light on the mechanisms underlying narrow-based gait in people with PD, first in order to understand why some people with PD walk narrow-based, but also to better understand the relation between trunk movements and mediolateral balance control during walking in general. In line with our hypothesis, we found reduced mediolateral XCoM excursions and trunk rotation but similar mediolateral MoS in people with PD with narrow-based gait compared to age-matched healthy controls. These findings imply that narrow-based gait in PD did not affect mediolateral MoS.

In line with the literature, we found reduced trunk rotation in people with PD.^27^ These reduced trunk movements are most likely related due to trunk rigidity, a prominent characteristic of PD.^27,28^ In addition to reduced trunk rotation in PD, we found a concurrent reduction in mediolateral XCoM excursions. This differs from two previous studies that focused primarily on anteroposterior dynamic stability in people with PD.^29,30^ These studies reported no differences in mediolateral CoM dynamics between PD and controls. In another study that explored how PD affects lateral stability control during walking, no difference in step width was found between people with PD and healthy controls.^31^ Yet, in contrast to the present study, these studies did not impose a fixed gait speed. As (mediolateral) CoM excursions are influenced by gait speed, group differences in CoM dynamics may have been obscured by the variable gait speeds in these previous studies.^29–32^ In addition, our study only included people with PD with a narrow-based gait, which could explain the differences with previous studies.

In line with a previous study from our group—in which healthy people walked comfortably and with reduced trunk movements (i.e., rotation and obliquity)—we confirm that walking with reduced trunk motion (trunk rotation in people with PD) coincides with reduced XCoM excursion and step width, while maintaining a similar MoS.^8^ One may argue that differences in cadence or step scaling are a possible mechanism to explain reduced XCoM excursions and step width.^33^ However, all participants in our study walked at similar gait speed, and we found no differences in cadence (and therefore also not in step length) between the PD group and controls.

As PD progresses (e.g., Hoehn&Yahr stage 3–4), or with existing co-morbidities (e.g., vascular cerebral lesions), mediolateral balance capacity may become affected, necessitating a larger MoS.^11,34^ Thus, to compensate for reduced mediolateral balance capacity,^34^ these patients may broaden their step width. A broad-based gait pattern is a common response of people with balance impairments (such as patients with ataxia^35^). Increasing the base of support, without altering the XCoM excursions to the same extent, will increase the MoS, which enables patients to compensate for the reduced balance capacity. The results of our beam condition confirm that people with PD are able to walk with a broad-based gait. Previous work in healthy people, however, has shown that this may come at the cost of gait efficiency.^36^

To further explore the relationship between XCoM excursions and step width, we evaluated whether an instruction to increase arm swing would result in increased trunk motions and XCoM excursions. To verify the idea that increased arm swing would increase trunk motion, XCoM excursions, and step width, we first evaluated this in the healthy controls. Indeed, exaggerated arm swing significantly increased these parameters while having no effect on mediolateral MoS. In contrast to the control group, exaggerated arm swing in PD yielded only very small increments in trunk obliquity and XCoM excursions. This may be a consequence of trunk rigidity, as trunk rotations only increased in the three less affected patients (i.e., those with the lowest MDS-UPDRS part III scores). Hence, in PD the arm swing condition failed to manipulate trunk movements and XCoM excursions sufficiently.

This study was not without shortcomings. To compare both groups in terms of trunk motion, XCoM, MoS, and step width, we have controlled for gait speed. The fixed gait speed of 0.6 m s^−1^ may have been rather slow for the control group. In addition, for some participants, walking on a dual-belt treadmill was an unfamiliar experience, leading to differences in gait compared to straight walking during daily life (particularly the people with PD tended to increase their step width slightly compared to straight walking during daily life).^37^ Hence, in terms of step width, the differences between PD and controls in this study may have been smaller than in real life. Importantly, we included people with PD who already exhibited a narrow-based gait. Hence, our findings do not generalize to the PD population at large but only to the PD population with a narrow-based gait. Lastly, as trunk rigidity may be at the basis of a narrow-based gait patters, the lack of a measure of trunk rigidity is a limitation. However, as far as we are aware, there is no validated measure for trunk rigidity.

In conclusion, we provide further evidence for a strong correlation between trunk motion and step width. In PD, reduced trunk motion may contribute to narrow-based gait, without affecting mediolateral MoS. However, as we could not substantially increase trunk motion in PD during the arm swing condition, future research is needed to confirm acausal relationship between reduced trunk motion and narrow-based gait in PD. Furthermore, future work may longitudinally assess trunk rigidity and trunk movements, step width and presence of mediolateral balance impairments to further test this conceptual framework.

## Supplemental Material

sj-docx-1-pkn-10.1177_1877718X241313333 - Supplemental material for Narrow-based gait in people with Parkinson's disease: Its mechanisms explored Supplemental material, sj-docx-1-pkn-10.1177_1877718X241313333 for Narrow-based gait in people with Parkinson's disease: Its mechanisms explored by Jamie AF Jansen, Tom JW Buurke, Lotte van de Venis, Vivian Weerdesteyn, Noël Keijsers and Jorik Nonnekes in Journal of Parkinson's Disease
