# Supplementary material for: Narrow-based gait in people with Parkinson's disease: Its mechanisms explored
Source: J Parkinsons Dis. 2025 Feb 2:1877718X241313333. Online ahead of print. doi: 10.1177/1877718X241313333 (PMC13347439; doi:10.1177/1877718X241313333)
Supplement: Supplementary file 1 [file sj-docx-1-pkn-10.1177_1877718X241313333.docx]

**Supplemental Material**

**Narrow-based gait in people with Parkinson’s disease: Its mechanisms explored**

**Supplemental Figure 1.** Comparison between baseline (grey) and the arm swing (red) condition in controls. Controls were able to increase their degree of arm swing, trunk rotation and obliquity, XCoM excursion and step width. MoS did not differ between conditions.
